# Longitudinal Analyses Indicate Bidirectional Associations Between Loneliness and Self-Rated Health in Adulthood

**DOI:** 10.1093/geroni/igab046.2067

**Published:** 2021-12-17

**Authors:** Deborah Finkel, Dianna Phillips, Chandra Reynolds

**Affiliations:** 1 Indiana University Southeast, New Albany, Indiana, United States; 2 University of California Riverside, Riverside, California, United States

## Abstract

Loneliness is a potent stressor that increases in prevalence with age in late life and has been linked with numerous adverse physical health outcomes and lower scores on measures of self-rated health (SRH). The association between loneliness and SRH is likely bidirectional—for example, experiencing loneliness may result in physiological changes that alter how individuals perceive their health, and worsening perceptions of one’s own health or mobility may act in an increasingly restrictive manner with respect to social interaction. Despite this, limited longitudinal work has examined temporal dynamics between loneliness and SRH. Recently completed harmonization of 9 loneliness items across three longitudinal twin studies of aging in Sweden resulted in sample of 1939 participants aged 40 to 98 at intake (mean age = 74.64) with up to 25 years of follow-up (mean = 7.63) across up to 8 waves (mean = 3.29). Univariate analysis indicated that SRH decreased with age up to age 82 and then leveled off, whereas loneliness continued to increase across the age span. Bivariate dual change score models were used to examine lead-lag relationships across time: which variable contributes to subsequent changes in the other variable. Results indicated a bi-directional relationship: loneliness does not increase after age 82 when SRH is included in the model, and SRH does not level off after age 70 when loneliness is included in the model. Thus, declining SRH may lead to reduced participation in social activities and also feelings of loneliness may intensify perceptions of poor health.

